# Bilateral 5 Hz transcranial alternating current stimulation on fronto-temporal areas modulates resting-state EEG

**DOI:** 10.1038/s41598-017-16003-2

**Published:** 2017-11-15

**Authors:** Aurora D’Atri, Claudia Romano, Maurizio Gorgoni, Serena Scarpelli, Valentina Alfonsi, Michele Ferrara, Fabio Ferlazzo, Paolo Maria Rossini, Luigi De Gennaro

**Affiliations:** 1 Department of Psychology, University of Rome “Sapienza”, Via dei Marsi 78, 00185 Rome, Italy; 2 IRCCS San Raffaele Pisana, Via della Pisana 235, 00163 Rome, Italy; 3 Department of Biotechnological and Applied Clinical Sciences, University of L’Aquila, Via Vetoio (Coppito 2), 67100 Coppito, L’Aquila, Italy; 4 Institute of Neurology, Catholic University of The Sacred Heart, Largo Agostino Gemelli 8, 00168 Rome, Italy

## Abstract

Rhythmic non-invasive brain stimulations are promising tools to modulate brain activity by entraining neural oscillations in specific cortical networks. The aim of the study was to assess the possibility to influence the neural circuits of the wake-sleep transition in awake subjects via a bilateral transcranial alternating current stimulation at 5 Hz (θ-tACS) on fronto-temporal areas. 25 healthy volunteers participated in two within-subject sessions (θ-tACS and sham), one week apart and in counterbalanced order. We assessed the stimulation effects on cortical EEG activity (28 derivations) and self-reported sleepiness (Karolinska Sleepiness Scale). θ-tACS induced significant increases of the theta activity in temporo-parieto-occipital areas and centro-frontal increases in the alpha activity compared to sham but failed to induce any online effect on sleepiness. Since the total energy delivered in the sham condition was much less than in the active θ-tACS, the current data are unable to isolate the specific effect of entrained theta oscillatory activity *per se* on sleepiness scores. On this basis, we concluded that θ-tACS modulated theta and alpha EEG activity with a topography consistent with high sleep pressure conditions. However, no causal relation can be traced on the basis of the current results between these rhythms and changes on sleepiness.

## Introduction

In the last two decades, a growing number of human studies has provided support to the idea that rhythmic Non-Invasive transcranial Brain Stimulation (NIBS), such as repetitive transcranial magnetic stimulation (rTMS), transcranial alternating current stimulation (tACS), and oscillatory transcranial direct current stimulation (o-tDCS), can influence brain activity, including the aspects related to sleep and arousal, via frequency-specific modulations of cortical activity.

Results from rTMS^1^ and o-tDCS^2^ studies converged in indicating that low frequency stimulations (<1 Hz) during sleep result in the enhancement of sleep-specific EEG patterns, such as slow waves, K-complexes and slow spindles^1,2^. According to the well-known role of the thalamo-cortical bidirectional projections in orchestrating these EEG patterns during sleep (for a review, see^3^), these findings suggest the possibility to modify sleep dynamics by mean of an external ‘top-down’ modulation of the thalamo-cortical network.

Moreover, findings from studies that applied cortical rhythmic stimulations during wakefulness suggest the possibility to interact with the ‘top-down’ control of sleep to increase sleep propensity. In other words, it could be ideally possible to trigger or facilitate the sleep onset process by influencing the cortical components of the sleep control system^4^. Along this line, a pioneer study showed that a stimulation in the theta range (6 Hz) of the subcallosal region and orbital gyrus of the frontal lobe through chronically implanted electrodes in unanesthetized cats resulted in cortical synchronization and light sleep^5^.

In humans, electrosleep and electroanesthesia have been studied since a long time^6,7^ (for a review, see^8,9^). More recently, it has been demonstrated that theta-burst stimulation protocols (TBS) with TMS, in which gamma-bursts (50 Hz) at the theta frequency (5 Hz) are applied, were able to trigger delta waves in the awake brain when supplied as intermittent pattern (iTBS)^10^. This stimulation produces a significant reduction of stage-2 latency in subsequent sleep, consistent with a more rapid sleep onset process, along with a significant improvement of sleep efficiency when supplied as continuous stimulation (cTBS)^11^. However, the stimulation did not affect objective measures of vigilance [i.e., maintenance of wakefulness test (MWT), and psychomotor vigilance task (PVT)].

Frase and colleagues^12^, instead, recently found that a bi-frontal anodal constant tDCS during pre-sleep wakefulness induces opposite effects: it enhances high frequency EEG activity and results in a reduction of total sleep time, i.e. a worsening in sleep continuity, without affecting subjective measures of tiredness.

Moreover, 0.75 Hz o-tDCS applied during wake resulted in local increases of slow oscillations and widespread increase of the theta EEG activity^13^. However, self-reported sleepiness measures were not affected by this type of stimulation^13,14^, suggesting that the induction of sleep-like slow frequency EEG activity does not necessarily affect the conscious sleep pressure at the subjective level.

Contrary to these findings, we recently found that a frontal anodal stimulation in the theta range (5 Hz o-tDCS) during wakefulness resulted in a significant enhancement of sleepiness level, correlated with stimulation-induced variations in delta activity in a narrow region near the stimulation electrode^15^. Since the well-known role of the theta rhythm as a marker of sleepiness during wakefulness^16–19^ and during early phases of spontaneous sleep onset^20–23^, the ability of the theta-stimulation compared to the slow-oscillatory tDCS (so-tDCS) in affecting self-reported sleepiness measures could be likely explained by the physiological meaning of theta activity in the awake brain EEG.

**Figure 1:**
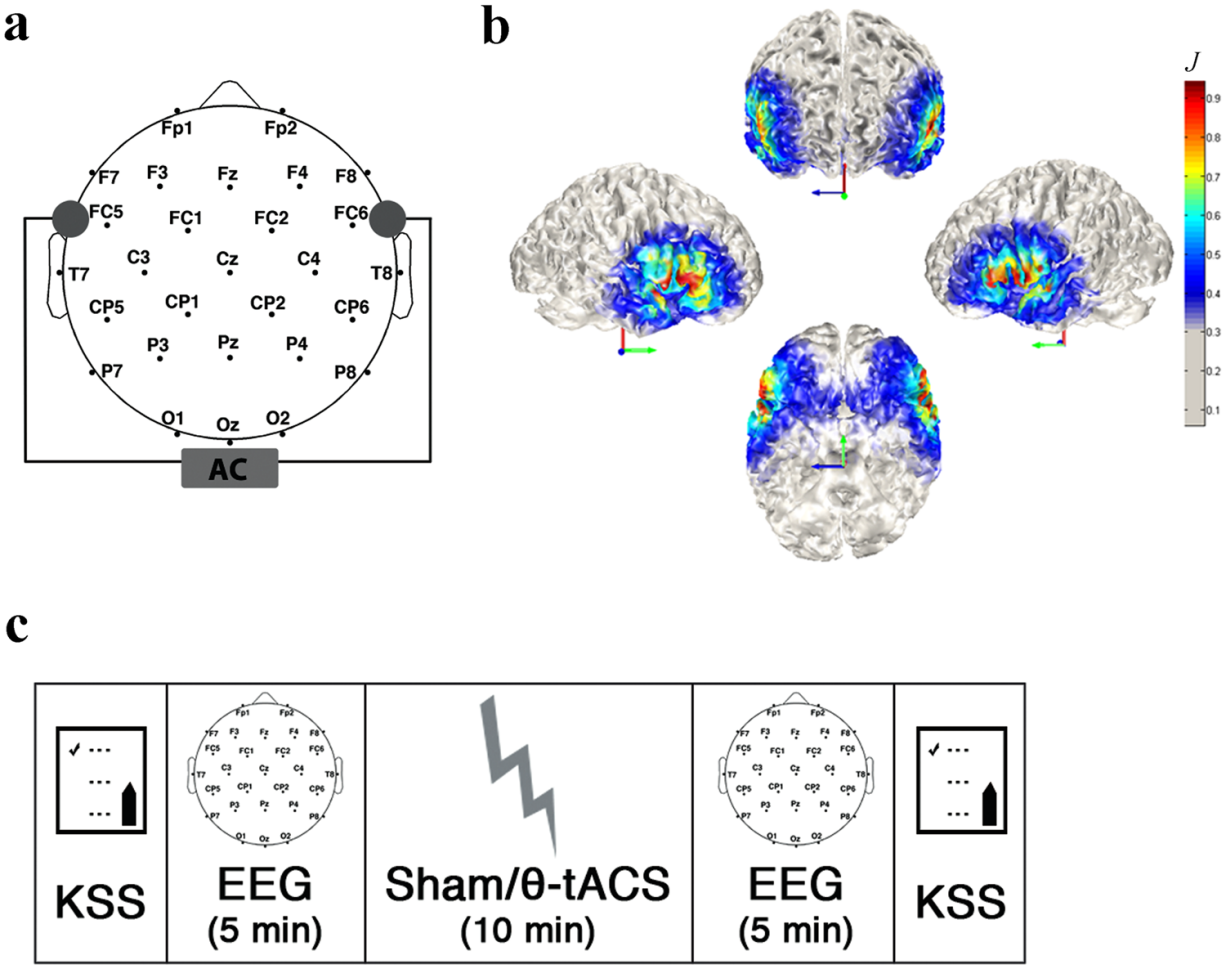
Experimental design. (**a**) EEG recording montage and stimulation electrodes montage (grey circles). (**b**) 3D finite element modeling of current density distribution with the bilateral fronto-temporal stimulation montage, using the COMETS toolbox for MATLAB^47^. (**c**) Experimental design (KSS: Karolinska Sleepiness Scale^40^; θ-tACS: 5 Hz transcranial Alternating Current Stimulation; AC: Alternating Current).

Nevertheless, the effects of the stimulation on EEG measures are characterized by a small magnitude^15^, likely due to the large distance between the two stimulation electrodes in the specific montage usually adopted, with the extra-cephalic reference placed on the deltoid muscle^24^. In the present study, therefore, we opted for a totally cephalic bipolar montage in which the current flow is mainly radial, therefore penetrating the brain structures, with the two stimulation electrodes placed on the left and right fronto-temporal areas (Fig. 1a). This type of montage has the highest ability for targeting deep brain structures like those in the mesial temporal lobes and the brainstem. We also explored effectiveness on a different target cortical areas with respect to previous TMS and tDCS/tACS studies aimed to similar purposes, which stimulated midline or dorsolateral frontal cortex^1,2,11,13,15,25,26^. Here, the electrodes placement allowed the bilateral stimulation of a region encompassing parts of the inferior and orbital gyri of the frontal lobe and parts of the anterior temporal lobe (Fig. 1b). Moreover, with this montage it was possible to suppose a direct or indirect (via cortico-subcortical connections) action of the stimulation on subcortical structures such as thalamus and hippocampus down to the brainstem, as demonstrated in electroanethesia in the early sixties (for a review, see^9^). Since these subcortical structures begin the synchronization process before the cortex during the spontaneous sleep onset^27–29^, a synchronizing action involving also these nuclei should make the stimulation more efficient in increasing sleep propensity.

Within the above theoretical frame, the aim of this study was to evaluate the possibility of enhancing sleep propensity by inducing synchronization in the cortico-subcortical networks involved in sleep onset with a 5 Hz tACS (θ-tACS) mainly targeting fronto-temporal areas (Fig. 1b). Consistently with the EEG dynamics characterizing the wake period immediately preceding sleep onset^20–23^, we expected a widespread increase in slow EEG rhythms, mainly in the delta and theta frequency bands, after tACS stimulation. On the basis of the dissociation between objective and subjective measures of sleep propensity reported by previous studies^12–15^, a secondary aim was to assess whether θ-tACS coherently affect EEG and self-rated measures of sleepiness.

## Results

### θ-tACS effects on cortical rhythms

**Figure 2:**
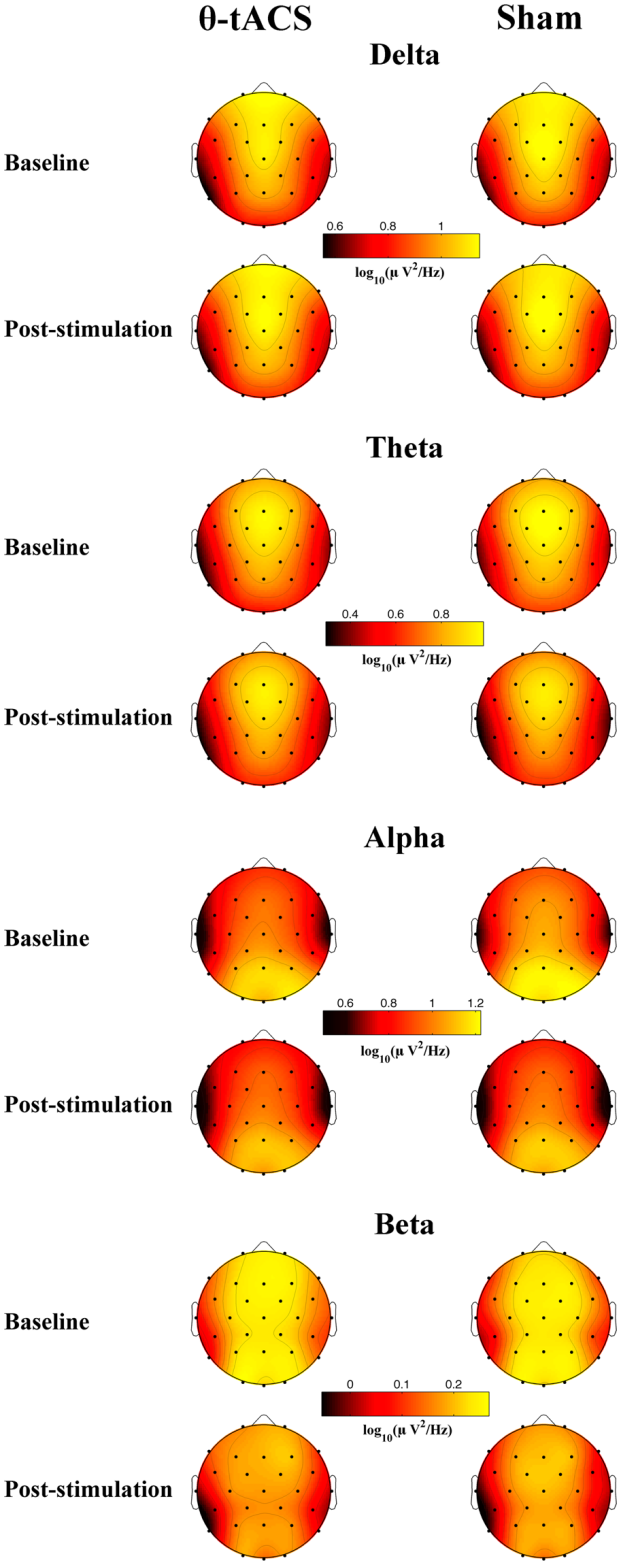
Topographic distribution of baseline and post-stimulation EEG spectral powers for θ-tACS and Sham conditions. Topographic maps of mean EEG spectral powers (log-trasformed) at the baseline and after stimulation associated with θ-tACS (first column) and sham (second column) protocols. Maps are plotted for the following frequency bands: delta (1–4 Hz), theta (5–7 Hz), alpha (8–12 Hz), beta (13–24 Hz). Values are colour coded and plotted at the corresponding position on the planar projection of the scalp surface and are interpolated (biharmonic spline) between electrodes. The maps are scaled between minimal and maximal values considering the two experimental conditions within each frequency band.

**Figure 3:**
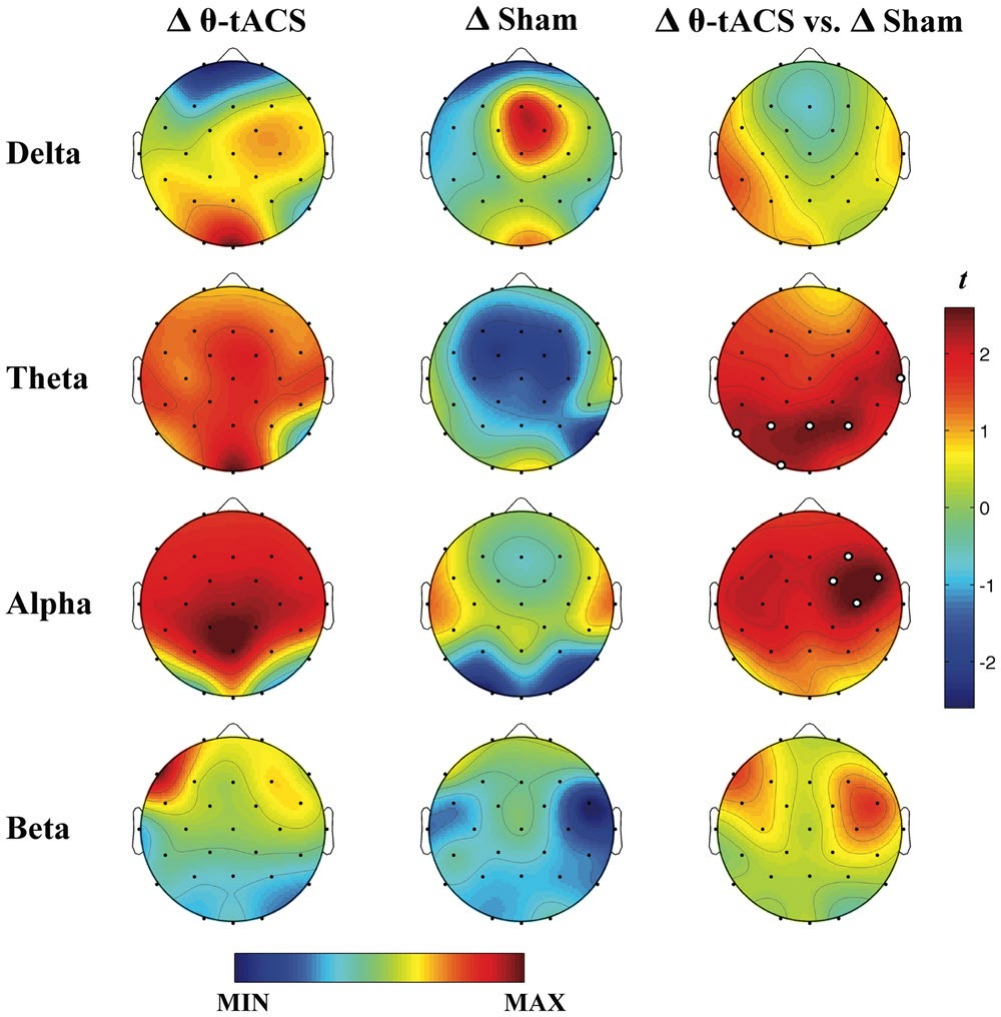
Cortical effects of θ-tACS: EEG frequency bands resolution. Topographic maps of mean EEG variations in spectral powers associated with θ-tACS (Δ θ-tACS, first column) and sham (Δ sham, second column) conditions and statistical maps of comparisons assessed by paired t-tests (Δ θ-tACS *vs*. Δ sham, third column). Maps are plotted for the following frequency bands: delta (1–4 Hz), theta (5–7 Hz), alpha (8–12 Hz), beta (13–24 Hz). Values are colour coded and plotted at the corresponding position on the planar projection of the scalp surface and are interpolated (biharmonic spline) between electrodes. The topographic maps (first and second columns) are scaled between minimal and maximal values considering the two experimental conditions within each frequency band. The statistical maps are scaled symmetrically according to the absolute maximal t-value across the statistical comparisons in all frequency bands. White dots represent significant statistical differences between θ-tACS and Sham conditions surviving the TFCE correction for multiple comparisons (p_*TFCE-corrected*_ < 0.05).

Baseline and post-stimulation EEG spectral powers associated with the θ-tACS and Sham conditions are shown in Fig. 2, while changes of EEG power induced by the two stimulation protocols, defined as difference in power between baseline and after-stimulation EEG, are shown in Fig. 3. The figure also details the results of corresponding statistical comparisons by paired two-tailed t-tests (Δ θ-tACS *vs*. Δ sham, n = 25). θ-tACS induced significant increases of the theta power compared to sham at O1 (t_24_ = 2.25, p_*TFCE-corrected*_ = 0.046), P3 (t_24_ = 2.31, p_*TFCE-corrected*_ = 0.038), P4 (t_24_ = 2.44, p_*TFCE-corrected*_ = 0.036), P7 (t_24_ = 2.21, p_*TFCE-corrected*_ = 0.046), Pz (t_24_ = 2.47, p_*TFCE-corrected*_ = 0.036), T8 (t_24_ = 2.41, p_*TFCE-corrected*_ = 0.036) EEG recording sites. θ-tACS also increased the alpha power at some right centro-frontal sites C4 (t_24_ = 2.58, p_*TFCE-corrected*_ = 0.034), F4 (t_24_ = 2.13, p_*TFCE-corrected*_ = 0.046), Fc2 (t_24_ = 2.25, p_*TFCE-corrected*_ = 0.036), Fc6 (t_24_ = 2.51, p_*TFCE-corrected*_ = 0.034). These effects remain significant also when correcting for the multiple comparisons through the false discovery rate (FDR; q-values < 0.04)^30^.

### 1-Hz resolution of θ-tACS cortical effects

**Figure 4:**
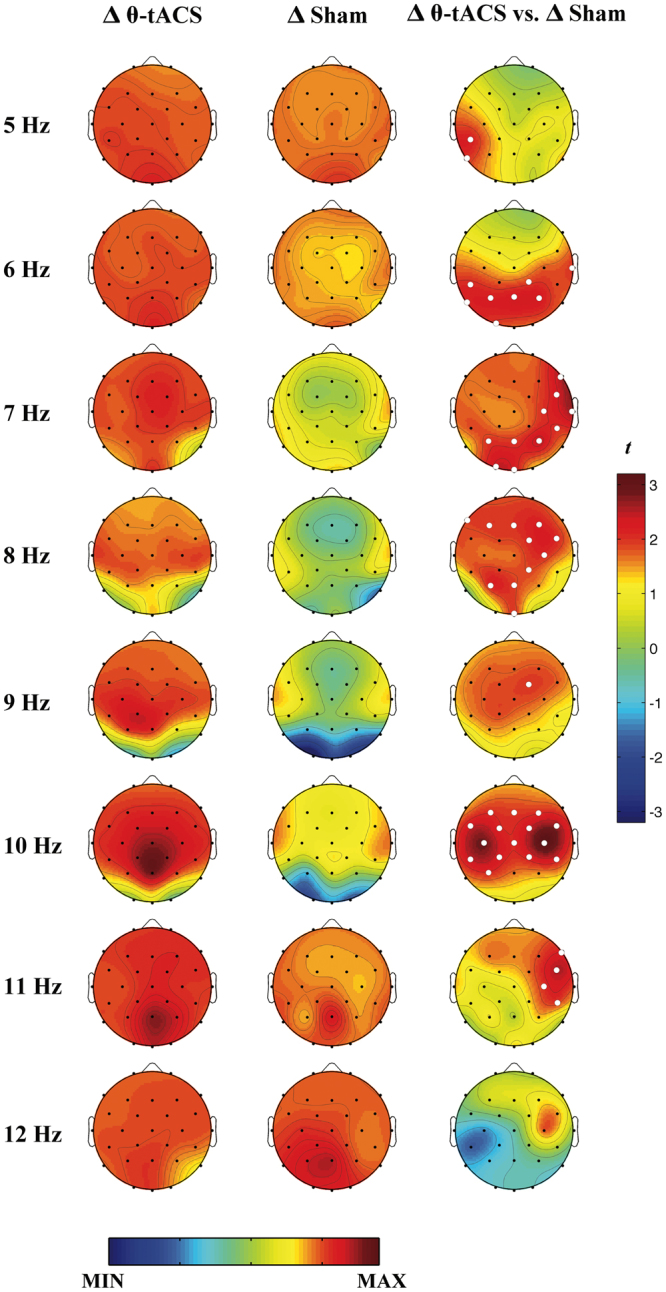
Cortical effects of θ-tACS: single-Hz frequency bin resolution. Topographic maps of mean EEG variations in spectral powers with 1-Hz resolution associated with θ-tACS (Δ θ-tACS, first column) and sham (Δ sham, second column) conditions and statistical maps of comparisons assessed by paired t-tests (Δ θ-tACS *vs*. Δ sham, third column). Maps are plotted in the theta and alpha frequency range (5–12 Hz) with a single-Hz resolution. Values are colour coded and plotted at the corresponding position on the planar projection of the scalp surface and are interpolated (biharmonic spline) between electrodes. The topographic maps (first and second columns) are scaled between minimal and maximal values across the two experimental conditions and all frequency bins. The statistical maps are scaled symmetrically according to the absolute maximal t-value across the statistical comparisons in all frequency bins. White dots represent significant statistical differences between θ-tACS and Sham conditions surviving the TFCE correction for multiple comparisons (p_*TFCE-corrected*_ < 0.05).

To better clarify at which frequency larger effects were induced, we replicated the analysis by paired two-tailed t-tests (Δ θ-tACS *vs*. Δ sham, n = 25) on the EEG power variations within the theta and alpha ranges (5–12 Hz) with a 1-Hz resolution. This analysis was aimed to assess whether the effect for the alpha activity could be considered as a tail of the theta effect, i.e. if it was focused on the lower frequency bins of the alpha band. The results are shown in Fig. 4. The statistical comparisons between the two experimental conditions within the theta range confirmed the presence of larger variations in θ-tACS compared with sham (p_*TFCE-corrected*_ < 0.05) at posterior areas that gradually shift at more anterior location as the frequency increases from 5 up to 8 Hz, the last bin in fact belonging to the alpha range in the band analysis. In the alpha range, the main result is represented by an extended effect of the stimulation at 10 Hz frequency bin, encompassing a large portion of the scalp with a bilateral peak at central areas (C3: t_24_ = 2.86, p_*TFCE-corrected = *_0.031; C4: t_24_ = 3.2, p_*TFCE-corrected = *_0.028).

### Self-rated sleepiness

**Figure 5:**
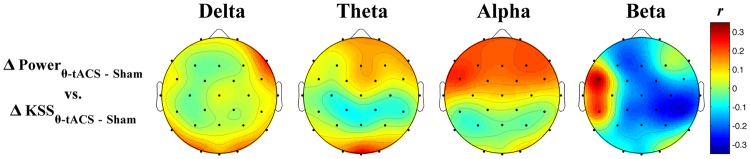
Correlations between changes in EEG power and subjective sleepiness. Topographic distribution of the correlation coefficients (Pearson’s r) between changes in subjective sleepiness and EEG power changes expressed as (Δθ-tACS – ΔSham) differences.

The repeated measures ANOVA on KSS scores (n = 25) reported a significant main effect of the Phase (F_1,24_ = 28.97, p = 0.000016), in the direction of a robust increase of sleepiness level from baseline to post-stimulation in both the sham (t_24_ = 4.45, p = 0.0002) and the active condition (t_24_ = 4.92, p = 0.00005). No significant main effect of the Stimulation or Phase × Stimulation interaction was observed (F_1,24_ < 1). Nevertheless, we evaluated if KSS score, expressed as difference between active and sham conditions, were related to EEG changes. No correlation was significant (r ≤ 0.35) for any band or scalp location (Fig. 5).

## Discussion

Our study was aimed at assessing the possibility to manipulate sleep propensity during wakefulness via a 5 Hz tACS mainly targeting fronto-temporal areas. Results confirm the possibility to modulate the cortical activity by non-invasive transcranial rhythmic stimulation of specific cortical networks. The θ-tACS on fronto-temporal areas results in a significant increase of the spectral power in the theta and alpha frequency bands compared with the sham condition. In particular, the differences in the theta band involved the posterior areas, while the effect of stimulation on the alpha band peaked at the centro-frontal areas, with a frequency-specificity at 10 Hz. Although our analyses demonstrate that θ-tACS modulated offline theta and alpha EEG activity but failed to induce any online effect on sleepiness. Although these cortical effects are consistent with a relatively increased sleep propensity in the active compared to the sham condition (see below), the self-reported measures of sleepiness (KSS) do not show significant differences between the two experimental conditions. Though, this is probably a ‘ceiling effect’ as a robust increase of sleepiness was also observed in the control condition, likely due to several interacting factors (e. g., time-of-day effects, post-lunch dip, eyes-closure during the whole session, in a comfortable position). This dissociation between objective and subjective measures of sleepiness confirms previous studies using rTMS^11^ or tDCS^12^ protocols, which have shown a potential to impose or entrain specific EEG rhythms. More in general, it raises once again the issue of a higher level of vulnerability of subjective measures of sleepiness to external and motivational factors^31^.

Larger variations of the spectral power in the theta band after the θ-tACS compared to the sham stimulation are consistent with the expected outcome of promoting the cortical synchronization linked with the sleep onset process. The analysis at a 1-Hz resolution showed that the phenomena induced by the stimulation involved not only the specific bin corresponding to the stimulation frequency (i.e., 5 Hz), but the whole theta frequency band (5–7 Hz) and the adjacent bin at 8 Hz (Fig. 4), suggesting the induction of a more global and physiological synchronizing effect rather than the simple entrainment of cortical oscillation at the stimulation frequency.

The posterior location of this effect (Fig. 3) suggests some speculations. When we become progressively drowsier before falling asleep or during a prolonged wakefulness, the frontal areas are usually the firsts to show an increase in EEG theta activity, which then gradually spreads more posteriorly, eventually involving the whole cerebral cortex. This has been observed at sleep onset^21,23^ and as a consequence of sleep deprivation^16,17,19,32^. Accordingly, we expected that any increase in sleep propensity due to the stimulation should be associated with a frontal increase in the theta power. A possible interpretation of the significant differences between the active and sham stimulation centred on posterior areas could be linked to the presence of a spontaneous increase of sleepiness level in both the experimental conditions, as the self-report measures actually showed. Therefore, the differences in the theta power at frontal areas were likely comparable between active and sham stimulation in that they were both linked to an increase in sleepiness. On the other hand, results show that θ-tACS induced more widespread changes encompassing also temporo-parieto-occipital areas, interpretable as a later stage within the process of a physiological sleep onset. In support of this interpretation, the increases in self-reported (KSS) and behavioural (PVT) sleepiness after sleep deprivation are mainly correlated with the theta power augmentation over the temporo-parieto-occipital areas^19^. Furthermore, in a study on spatio-temporal EEG pattern during the transition to sleep, Marzano and colleagues^23^ found that, although sleep onset induces a significant increase of spectral power in the theta range (5–7 Hz) involving the whole cortical topography, the largest effect was centred on parieto-occipital areas^23^. Why these changes also involve parietal areas? We have not a univocal explanation. Taking into consideration that the clusters of significant electrodes include also locations close to the site of stimulation, we may speculate that our effects are likely involving several nodes of a widespread network^33^ mediated at both cortical and subcortical levels, and different contribution of subcortical or cortical effects may have increased the theta activity and alpha activity in different cortical areas. Actually, the results reported in Fig. 4 show that the effects for the theta activity involve a cluster of electrodes whose contours include temporal, parietal and occipital areas with respect to the lower bound of the frequency range. Then, these changes gradually shift at more anterior locations across faster frequencies (i.e., at 8 Hz). This pattern of changes partly coincides with that of sleep onset^23^ and the gradual involvement of the dedicated network and related nodes.

Spontaneous sleep onset is a complex and dynamic process involving cortical and subcortical areas, at different times. It dynamically changes in space (i.e., brain areas) and time (i.e., a specific point in the transition). Also, the current five-minute recordings of post-stimulation resting EEG intrinsically convoy information on a process varying in space and time, by averaging a quite long time interval. Hence, finding different spatial differences for different bands may also reflect physiological processes at different times in a network configuration. Although the magnitude of the variations is not comparable between the two conditions (i.e, spontaneus sleep onset and the current resting EEG), as here we compared EEG changes in awake subjects after a 10-min stimulation instead of sleep *vs*. wake EEG, the topographical- and frequency-specificity of the stimulation effects is consistent with the achievement of a more advanced phase of the sleep onset transition in θ-tACS compared to the sham condition. At this time and with this kind of experimental procedures, we can only suggest that it mostly resembles similar changes occurring during spontaneous waking-sleep transition.

At variance with the effect on the theta band, the centro-frontal increase of the alpha activity was unexpected. Indeed, the period preceding sleep onset, from wake through sleep stage 1, is usually characterized by a progressive decrease in spectral power of the alpha band until the entrance in sleep stage 2^20,22,23^. Only after this point, representing the most reliable boundary between wakefulness and sleep^22^, the alpha power starts to increase on the frontal areas^21,23^ along with an anterior shift in the alpha sources^34^ and with an inversion in the direction of the cortical functional coupling, from a posterior-to-anterior in the pre-sleep period to an anterior-to-posterior directionality after sleep onset^35^. According to these dynamics, the frontal alpha increase should be considered as a feature characterizing a sleep state, rather than pre-sleep wakefulness, and its occurrence in a waking EEG is therefore difficult to interpret. However, some indications suggest that an increase of alpha power over the anterior derivations during wakefulness is associated with a condition of forced increase of sleep pressure during sleep deprivation/restriction protocols. In particular, significant increases in the alpha power at the centro-frontal regions during wakefulness were reported after a 40-h sleep deprivation protocol^19^. Furthermore, the shift to the anterior-to-posterior directionality of functional cortical coupling in the alpha band was found to be already present during the pre-sleep period after selective slow wave sleep deprivation^35^.

According to these findings, our increase of centro-frontal alpha power after θ-tACS could be seen as the intrusion during wakefulness of a sleep-like activity pattern, again indicating a high sleep pressure and the consequent difficulty of the participants to remain awake.

Results from a recent study on behavioural microsleep-related EEG activity in non-sleep deprived awake subjects^36^ support this interpretation. Behavioural microsleeps represent unintentional brief lapses of responsiveness (1–15 s) during a task associated with behavioural signs of sleep, such as eyelid closure and head-nodding^37^. These events are characterized by increases in the theta and alpha EEG power compared to a voluntary eyes-closed condition^36^ and by a transient bilateral thalamic deactivation (or decrease of thalamo-cortical bidirectional connectivity) considered to be central to the loss of arousal associated to this state^38^. These types of lapses are very frequent in high sleep pressure condition, such as in case of sleep restriction or deprivation^39^, albeit they can be easily found also in people whose sleep propensity is not manipulated, while they are performing a monotonous task^36,37^. In the current study, the stimulation effect at 10 Hz (Fig. 4) showed a topographic alpha distribution that actually resembles the one characterizing the alpha power increase during microsleeps reported by Jonmohamadi *et al*.^36^. Moreover, they identified different sources for the alpha activity linked to voluntary eyes-closed condition respect to the alpha activity linked to microsleeps: the first originates in the bilateral posterior cingulate cortex, while microsleep-related alpha sources have been localized in the anterior temporal lobe (superior and inferior) and the hippocampi^36^. Given the stimulation sites adopted in the current study at bilateral fronto-temporal areas, the bilateral centro-frontal increase in alpha power after θ-tACS, together with the posterior increase in theta power could be seen as indications of microsleep occurrence triggered by the rhythmic stimulation of their cortical source respectively in the anterior temporal lobes and frontal orbital areas.

Some limitations of the present experimental protocol must be acknowledged. Although the choice of a sham stimulation, in which the current injection stops after few seconds, made the participants blind about the experimental conditions, it does not allow to control for the effect of the current injection itself. According with this notion, a control condition with a high frequency fronto-temporal stimulation or transcranial Random Noise Stimulation (tRNS) could have confirmed the dependency of the cortical effects on the stimulation frequency. Actually, our analyses demonstrate that 5 Hz-theta tACS modulated offline theta and alpha EEG activity but failed to induce any online effect on sleepiness. The lack of a subjective impact was found even when sleepiness scores under active theta tACS were compared to those under sham tACS, a control condition which was only active for the 10 second current ramp up and ramp down periods, hence delivered much less total energy than the active 5 Hz-theta tACS used as active condition in the study. A comparison between these two tACS conditions would have been in any case unable to isolate the specific effect of entrained theta oscillatory activity *per se* on sleepiness scores. An accurate comparison able to single out the impact of theta oscillation frequency would require a comparison of the former with an active tDCS/tACS/tRNS pattern carrying a similar amount of total current over time, but delivered with a temporal dynamic different than a pure 5 Hz regular rhythm.

A second issue concerns the electrodes montage adopted in this study. Meanwhile, following the rules of Good medical practice for clinical trials with new drugs, a “placebo” condition (that is identical montage without stimulation) was preferred.

As described in the introduction, the electrodes placement has been determined in order to maximize the stimulation effect on subcortical structures involved in the spontaneous sleep onset. However, a tACS applied with this setup results in an anti-phasic stimulation of the two hemispheres. Although the results seem to support the hypothesized induction of synchronization within the cortical-subcortical networks involved in sleep-wake regulation, we cannot exclude that the anti-phasic stimulation of the fronto-temporal areas might have affected in some way the current findings. Actually, the neuronal correlates of the transition from wakefulness to sleep remains unclear, mainly due to the difficulty in capturing its inherent dynamics. A recent study has simultaneously explored EEG theta/alpha neurofeedback combined with fMRI to reveal state-dependent neural activity during wake-to-sleep transition^33^. Based on the known EEG signature of theta power increases over alpha, a “transition” point was marked and the fMRI activation was considered before and after this point. Decreased fMRI activity mainly characterized sensory gating related regions (e.g., medial thalamus); in parallel, theta EEG modulation corresponded with increased activity in limbic and autonomic control regions (e.g., hippocampus, cerebellum vermis, respectively) and with increased variance in the posterior salience network (e.g. posterior insula, posterior cingulate cortex), while alpha EEG modulation corresponded to reduced fMRI activity within the anterior salience network (e.g., anterior cingulate cortex, anterior insula;). Frontotemporal scalp stimulation site as in our protocol probably represents one of the “entry doors” to the several nodes representing the background neural network behind the wake-to-sleep transition. Modulation of the theta-dependent relays is probably mediating also alpha-dependent relays modulation.

While the frequency of ds-TACS is matched, the phase of stimulation is either identical (in-phase stimulation) or opposite (anti-phase stimulation) in the two cortical target areas. In-phase stimulation is thought to synchronize the endogenous oscillations while anti-phase stimulation is thought to desynchronize neural oscillations in the two areas^40^. Several experimental conditions utilizing tACS resulted effective when in-phase, but in selective situations it was only found effective when applied with 180° phase difference between hemispheres (anti-phase), as compared to in-phase stimulation with 0° phase difference^41,42^.

How this factor may have affected our results has not a univocal interpretation. In our opinion, this would not necessarily be in contradiction with our hypothesis of a synchronization within the cortical-subcortical networks involved in sleep-wake regulation since the nodes of the network are situated at different depth and therefore impacted with different phase of the alternating current. As a relevant example, the hippocampus shows EEG synchronization (as expressed by occurrence of sleep spindles) several minutes before neocortical sleep onset^29^. In the meantime, inter-hemispheric hippocampal coherence in the low-frequency range is lower in NREM sleep as compared to REM sleep and presleep wakefulness^43^. According to this speculation, an anti-phasic stimulation may have affected the inter-hemispheric hippocampal synchronization, but this is not at all in contradiction with the process of falling asleep.

In conclusion, a bilateral fronto-temporal θ-tACS induced a cortical pattern characterized by EEG features that are usually peculiar of sleep-deprivation conditions and microsleep occurrence, that is situations in which a high sleep pressure results in the intrusion of sleep-like activity during wakefulness. Although θ-tACS modulated the theta and alpha EEG activity with a topography consistent with high sleep pressure conditions, no causal relation can be traced on the basis of the current results between these rhythms and changes on sleepiness. can be traced on the basis of the current results. In this sense, further study studies should investigate on the possibility that the cortical effects of the stimulation may directly modulate the cortical-subcortical networks involved in sleep-wake regulation via transcranial stimulations and may affect also subjective sleepiness. It would be of considerable interest to assess, in subjects who are free to sleep, whether this type of stimulation is also able to coherently affect the subsequent sleep onset dynamics, in terms of sleep latency, sleep stability in the first portion of the night, and heightened homeostatic pressure (i.e., a steeper build-up of slow-wave activity).

## Methods

### Participants

Twenty-five healthy subjects (15 males and 10 females) aged between 18 and 35 years (mean age 25 ± 3.4 years) participated in the study after providing written informed consent. All participants were medication-free. Presence or history of epilepsy, neurological or psychiatric disorder and intracranial metal implants form the exclusion criteria.

The study was approved by the Institutional Ethics Committee of the Department of Psychology of University of Rome Sapienza and of the IRCCS San Raffaele Pisana, and was conducted in accordance with the Declaration of Helsinki.

### Experimental design

During the week before the experimental sessions, participants were asked to keep constant their wake-sleep cycle by sleeping about 7 h per night with a regular schedule, and to fill out a daily sleep log in order to control their compliance.

In the morning of the experimental sessions, they were not allowed to consume coffee, tea, chocolate, or any kind of neuroactive drugs.

The experimental procedure is illustrated in Fig. 1c. Each subject, blind to the type of stimulation, participated in two experimental sessions, a real stimulation condition (θ-tACS) and a sham condition, at least 1 week apart. The order of the stimulation conditions was balanced across subjects.

The timeline of the experimental session was the same regardless of the stimulation condition. Subjects arrived at the laboratory at 12:00 h. They had lunch at 12:30 h and then underwent application of EEG and stimulation electrodes. The experimental session started at 14:00 h, when the participant was asked to sit relaxed on a comfortable chair in a soundproof, temperature-controlled, and electrically shielded room with constant dim light.

Baseline EEG was recorded in a resting eyes-closed condition for 5 minutes. During recordings, subjects were asked to relax and reduce the flow of mental activity by imagining of fixating a point on the wall in front of them. The EEG signals were continuously monitored. Since the expected result of the stimulation was the increase in sleep propensity, during the online monitoring the occurrence of sleepiness signs consistent with a drowsy wakefulness (e.g. slow eyes movements or predominance of cortical activity at theta frequency) were allowed. Only when explicit signs of sleep intrusion were detected (e.g., K-complexes), the subject was addressed by the experimenter and asked to respond. The incidence of these episodes (10 and 7, in the active and sham conditions respectively) was not different between conditions (*χ* ^2^ = 0.79; p = 0.37).

θ-tACS or sham stimulation was then applied for 10 minutes. Subjects were asked to continue keeping their eyes closed also during the stimulation phase. Immediately after the end of the stimulation protocol, 5 minutes of post-stimulation EEG was recorded.

To evaluate if the possible stimulation effects on the EEG were associated with coherent variations of subjective sleepiness, self-reported measures of sleepiness were collected just before the start of baseline EEG recording and immediately after the end of post-stimulation EEG recording.

### EEG recordings

The EEG signals were recorded from sintered Ag–AgCl electrodes mounted in an elastic cap (Easycap, Falk Minow, Munich) at C3, C4, Cp1, Cp2, Cp5, Cp6, Cz, F3, F4, F7, F8, Fc1, Fc2, Fc5, Fc6, Fp1, Fp2, Fz, O1, O2, Oz, P3, P4, P7, P8, Pz, T7 and T8 locations of the international 10–10 system with linked mastoid references (Fig. 1b). Horizontal eye movements were detected by recording electro-oculograms (EOGs), and the electromyogram (EMG) was recorded by two submental electrodes for off-line artefacts detection. The ground electrode was placed anterior to Fz, at Fpz. Electrodes resistance was kept below 5 kΩ. Signals were amplified using the BrainAmp MR plus system (Brain Products GmbH, Gilching) and recorded with Brain Vision Recorder (Version 1.10, Brain Products GmbH, Gilching) at a sampling rate of 250 Hz (0.1 μV steps resolution). Raw EEG data were high-pass filtered with time constant of 1 s and low pass filtered at 70 Hz. The interference from the electricity network was eliminated by a notch filtering at 50 Hz (band-width of 5 Hz, symmetrical around to the 50 Hz frequency). The filters were implemented as phase shift-free Butterworth filters. EEG data were transferred to a computer by a fiber-optic cable and digitally stored on hard disk for further offline analyses.

### Electrical stimulation

In both the experimental conditions, the stimulation was applied via two conductive-rubber circular electrodes (diameter: 1.2 cm) placed in sponges saturated with high conductivity gel and connected to a battery-operated stimulator system (BrainSTIM, EMS medical). The two stimulation electrodes were placed on right and left fronto-temporal areas, between F7 (F8) and T7 (T8) of 10–10 system (Fig. 1a).

In θ-tACS condition, a sinusoidal alternating current with 5 Hz frequency and intensity ranging from 0 mA up to 0.6 mA was applied for 10 min (10 sec ramp in/out). In the sham condition, the stimulation setting was identical to the one adopted for θ-tACS, but the stimulator was turned off after 10 sec in order to maintain the same tingling sensation that subjects refer only at the beginning of the ‘real’ stimulation procedure.

No phosphenes sensations or adverse effects of the stimulations were reported by participants, but a slight and short lasting tingling under the stimulation electrodes. Participants noticed no differences among the active and sham conditions, as assessed by a post-experiment debriefing.

### Self-reported measures of sleepiness

The self-reported levels of sleepiness were collected with the Karolinska Sleepiness Scale (KSS^44^); at the beginning and at the end of the two experimental sessions. KSS is a nine-points rating scale, ranging from 1 up to 9 respectively corresponding to “Very alert” and “Very sleepy, fighting sleep”. Participants were asked to rate their sleepiness level on the basis of how they felt during the previous 5 minutes.

### Resting EEG analysis

Ocular and muscle artefacts in the EEG recordings were excluded by offline visual inspection of 2 sec epochs. Power spectra of the artefact-free epochs were computed by a Fast Fourier Trasform (FFT) routine for the 28 scalp locations in the 0.5–29 Hz range (1-Hz bin resolution except for the 0.5–1 Hz bin) and then averaged across epochs.

Data analysis was performed using the software package MATLAB 7.13 (The Math Works, Inc., MA, USA) and its statistics toolbox.

The spectral power values for the frequency bins were then averaged across the canonical EEG bands: delta (1–4 Hz), theta (5–7 Hz), alpha (8–12 Hz), beta (13–24 Hz).

Stimulation-related variations in EEG power were calculated for each frequency band and cortical derivation as difference in power between baseline and after-stimulation EEG and compared between the two experimental conditions by paired two-tailed t-tests (Δ θ-tACS *vs*. Δ sham). The significance level was corrected for multiple comparisons using threshold-free cluster enhancement (TFCE), a permutation-based approach that allows the significance to take into account not only the data point’s statistical intensity but also the contribution of both the spatial extent and the magnitude of the supporting neighbouring channels and frequency bins^45,46^. The correction was calculated by the free MATLAB statistical toolbox by Mensen (https://github.com/Mensen/ept_TFCE-matlab). Only TFCE-corrected p < 0.05 were reported.

### Subjective sleepiness analysis

To assess the possible effect of the stimulation on perceived sleepiness, KSS scores relative to before and after the stimulation in the two experimental conditions were compared by two-ways repeated measures analysis of variance (ANOVA) with Phase (before *vs*. after stimulation) and Condition (θ-tACS *vs*. sham) as within-subject factors. Post-hoc tests have been carried out by paired t-tests (p ≤ 0.05).

### Data Availability

The datasets analysed during the current study are available from the corresponding author on reasonable request.
